# Gene amplification in human cells knocked down for RAD54

**DOI:** 10.1186/2041-9414-2-5

**Published:** 2011-03-18

**Authors:** Aurora Ruiz-Herrera, Alexandra Smirnova, Lela Khouriauli, Solomon G Nergadze, Chiara Mondello, Elena Giulotto

**Affiliations:** 1 Dipartimento di Genetica e Microbiologia "Adriano Buzzati-Traverso", Università di Pavia, Via Ferrata 1, 27100 Pavia, Italy; 2 Istituto di Genetica Molecolare, CNR, Via Abbiategrasso 207, 27100 Pavia, Italy; 3 Departament de Biologia Cel.lular, Fisiologia i Immunologia and Institut de Biotecnologia i Biomedicina (IBB), Universitat Autònoma de Barcelona, 08193, Campus Bellaterra, Barcelona, Spain

## Abstract

**Background:**

In mammalian cells gene amplification is a common manifestation of genome instability promoted by DNA double-strand breaks (DSBs). The repair of DSBs mainly occurs through two mechanisms: non-homologous end-joining (NHEJ) and homologous recombination (HR). We previously showed that defects in the repair of DSBs *via *NHEJ could increase the frequency of gene amplification. In this paper we explored whether a single or a combined defect in DSBs repair pathways can affect gene amplification.

**Results:**

We constructed human cell lines in which the expression of RAD54 and/or DNA-PKcs was constitutively knocked-down by RNA interference. We analyzed their radiosensitivity and their capacity to generate amplified DNA. Our results showed that both RAD54 and DNA-PKcs deficient cells are hypersensitive to γ-irradiation and generate methotrexate resistant colonies at a higher frequency compared to the proficient cell lines. In addition, the analysis of the cytogenetic organization of the amplicons revealed that isochromosome formation is a prevalent mechanism responsible for copy number increase in RAD54 defective cells.

**Conclusions:**

Defects in the DSBs repair mechanisms can influence the organization of amplified DNA. The high frequency of isochromosome formation in cells deficient for RAD54 suggests that homologous recombination proteins might play a role in preventing rearrangements at the centromeres.

## Background

Different pathways, mainly controlling either the cell cycle in response to DNA damage or the repair of the damage itself, maintain genome stability in mammalian cells. Mutations in genes implicated in these pathways cause genetic lesions that can give rise to cellular transformation. Gene amplification, the increase in the copy number of a portion of the genome, is a common manifestation of genome instability in tumour cells and an important mechanism of oncogene activation as well as drug resistance, since it leads to over-expression of relevant genes. Amplification of DNA sequences containing cancer genes has been described in several types of solid tumours and lymphomas [1,2]. The fact that gene amplification has never been detected in cells of normal origin [3,4] suggests that either control mechanisms that prevent the occurrence of gene amplification are active (such as the p53-mediated damage-sensing pathway), or cells carrying gene amplifications do not survive.

Cytogenetic manifestations of amplified DNA include self-replicating extrachromosomal elements called double minutes (DMs), amplified regions on a single chromosome (homogeneously staining regions, HSRs) or amplified regions distributed throughout the genome [5]. The existence of specific regions of the genome that are hotspots for amplification in cancers with similar cell of origin suggests that they contain genes relevant for tumour formation and progression [6,7]. In addition, the genomic context where the amplified DNA is embedded [8] and its proneness to breakage [9] seem to contribute to the propensity to amplify of specific genomic territories. Moreover, the instability of amplified DNA further increases the extent of amplification. A large body of evidence indicates that DNA double-strand breaks (DSBs) can promote gene amplification through different processes such as successive breakage-fusion-bridge (BFB) cycles, unequal sister chromatid exchange, rolling circle replication or fold-back priming (for a review see [10]). Mammalian cells repair DSBs through two main mechanisms: (1) homologous recombination (HR), which requires large regions of homology, and (2) non-homologous end joining (NHEJ), which does not require extended homologies [11]. NHEJ is an error prone process that dominates during the G1 to early S phase of the cell cycle whereas HR is mainly used in the late S and G2 phases. The NHEJ pathway requires the activity of several proteins, including the DNA-PK complex, which is composed of a heterodimeric subunit with DNA end-binding activity (Ku) and a catalytic subunit, the DNA-dependent protein kinase (DNA-PKcs). The Ku proteins bind the ends of the DSB and recruit DNA-PKcs, whose kinase activity is essential for the activation of other repair factors. DNA-PKcs is therefore a key player in NHEJ and cells defective in the DNA-PKcs gene are hypersensitive to ionizing radiations [12-14]. In addition to DNA breaks, DNA-PKcs binds to telomeres and is involved in telomere maintenance; in fact, defective cells show an increased frequency of telomeric fusions [15-17]. Homologous recombination is also a complex mechanism requiring several proteins among which, RAD51 and RAD54 represent the key players. These proteins are members of the RAD52 epistatic group of genes that codify the enzymes implicated in the homologous recombination process and the repair of DSBs [18]. RAD51 is the recombinase that recognizes the region of homology and promotes strand exchange. The RAD54 protein is a dsDNA-dependent ATPase that interacts physically and functionally with RAD51 performing several important functions in HR; it translocates along the dsDNA inducing topological changes, binding Holliday junctions and driving their migration (for a review see [19]). The interaction of RAD54 with different repair proteins during the HR process indicates that it is an important player in this pathway [20,21]. Moreover, RAD54 defects can cause sensitivity to ionizing radiations [22].

The first line of evidence showing a link between gene amplification and DSBs repair mechanisms was obtained by Mondello and collaborators [23], who showed that a defect in DNA-PKcs increases the frequency of gene amplification by one order of magnitude both in Chinese hamster cells and in immortal mouse embryonic fibroblasts. In addition, pre-treatment of DNA-PKcs deficient cells with ionizining radiation further increases the frequency of the process [24]. These observations have been confirmed in human cells in which DNA-PKcs expression was constitutively inhibited by RNA interference [14]. These cells are more radiosensitive and prone to gene amplification than parental cell lines. A defect in the DNA-PKcs may facilitate gene amplification by delaying the joining of broken ends, by altering the equilibrium between NHEJ and HR or by increasing the frequency of telomeric fusions. A defect in the ATM pathway also causes an increased propensity to gene amplification [25]. DNA-PKcs and ATM defective cells possess the so called "amplificator" phenotype [26]. On the contrary, immortalized mouse embryonic fibroblasts derived from animals knocked-out for *mTERC *(the telomerase RNA component) did not show amplification capacity [27], suggesting that telomerase may be required for the stabilization of chromosomes carrying amplified DNA. In the light of these data, we can speculate that altered expression of other genes that participate in processes such as repair, recombination, cell cycle checkpoint control and telomere maintenance could affect the propensity or permissiveness to gene amplification.

In this paper we aimed at exploring whether a single or a combined defect in the homologous recombination and non-homologous end joining processes can affect gene amplification. To this purpose, we constructed human cell lines, derived from HeLa cells, in which the expressions of RAD54 and/or DNA-PKcs were constitutively knocked-down by RNA interference (KD cells). We analyzed the radiosensitivity of the different cell lines, together with their amplification ability. Moreover, we investigated the cytogenetic organization of the amplicons detected in clones isolated from cells knocked-down for the different functions. Our results suggest that defects in the DSBs repair mechanisms can influence the organization of amplified DNA.

## Results

### Construction of HeLa cell lines with stable inhibition of RAD54 and/or DNA-PKcs expression

To inhibit the expression of RAD54, HeLa cells were transfected with the plasmids shRAD54p-1, shRAD54p-2, shRAD54p-3 or shRAD54p-4, each containing the neomycin resistance gene and different oligonucleotides for the production of shRNA against human *RAD54*, or with the plasmid p-scrambled, containing an oligonucleotide that is not homologous to any human gene. Pools of G418 resistant clones were isolated and the level of *RAD54 *expression was measured by immunofluorescence using an antibody specific for human RAD54. As expected, the plasmid containing the scrambled sequence did not induce any reduction in *RAD54 *expression; plasmids shRNAp-1 and shRNAp-2 did not produce a detectable effect on *RAD54 *expression while plasmids shRNAp-3 and shRNAp-4 caused a reduction in the levels of the protein (results not shown).

**Figure 1:**
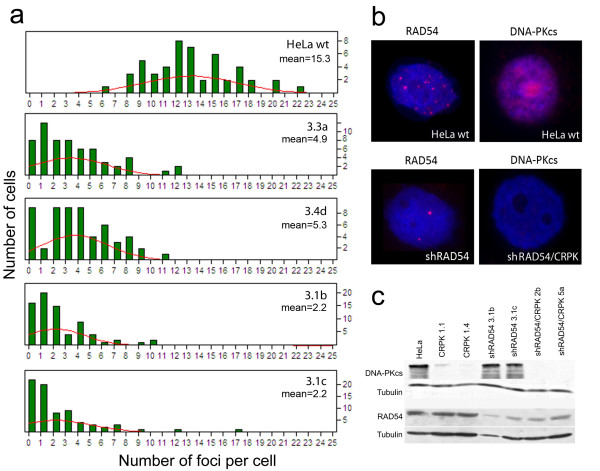
**Inhibition of RAD54 and DNA-PKcs expression in cell lines constitutively expressing shRNA**. Indirect immunofluorescence analysis of RAD54 and DNA-PKcs expression in cell lines constitutively expressing shRNA. (**a**) Distribution of the number of RAD54 *foci *detected in 50 cells of the HeLa parental cell line and in four clones knocked down for RAD54 (clones 3.3a, 3.4d, 3.1b and 3.1c). The number of foci per cell (X-axis) and the number of cells (Y-axis) are reported. Solid lines in red represent Normal distributions. The mean number of *foci *per cell is indicated for each cell line. (**b**) Examples of immunofluorescence using antibodies against the human RAD54 (left section of the panel) and the human DNA-PKcs (right section of the panel) proteins on the clones shRAD54 3.3a and shRAD54/CRPK 2b. In both cases the HeLa parental cell line was used as positive control. (**c**) Western blot analysis of DNA-PKcs and RAD54 expression in single and double knocked down clones. Tubulin was used as loading control.

Eleven clones containing the shRAD54p-3 and four clones containing the shRAD54p-4 plasmids were isolated and *RAD54 *expression was measured by immunofluorescence (Figure 1). In four out of the eleven clones containing the shRAD54p-3 plasmid (shRAD54 3.3a, shRAD54 3.4d, shRAD54 3.1b and shRAD54 3.1c) the expression of *RAD54 *was reduced (Figure 1a): a mean of ~15 RAD54 foci per cell was detected in parental HeLa cells while ~5 or less RAD54 foci per cell were detected in these clones. In none of the four clones containing the plasmid shRAD54p-4 inhibition of *RAD54 *expression was observed (data non-shown). The two clones (shRAD54 3.1b and shRAD54 3.1c) showing the lowest number of RAD54 foci (an average of 2.2 foci/cell, Figure 1a) were chosen for the long-term experiments required for radiation sensitivity and gene amplification analysis. In these clones, the residual level of the RAD54 protein, measured by western blotting, was about 40% (Figure 1c).

In order to obtain HeLa cell lines in which both *RAD54 *and *DNA-PKcs *expression were inhibited, the shRAD54 3.1b clone was transfected with the plasmid vector pCRPK1, which contains the puromycin resistance gene and an oligonucleotide for the expression of shRNA against DNA-PKcs [14]. Thirteen puromycin resistant clones were isolated and the level of *DNA-PKcs *expression was measured by immunofluorescence using an antibody specific for human DNA-PKcs. The two clones (shRAD54/CRPK 2b and shRAD54/CRPK 5a) with the greatest reduction in DNA-PKcs levels were chosen for further experiments. In these clones, DNA-PKcs was undetectable both by immunofluorescence and western blotting (Figure 1b and 1c) and RAD54 levels were similar to those observed in the single KD parental cell line shRAD54 3.1b (Figure 1c).

### Cellular sensitivity to ionizing radiation

**Figure 2:**
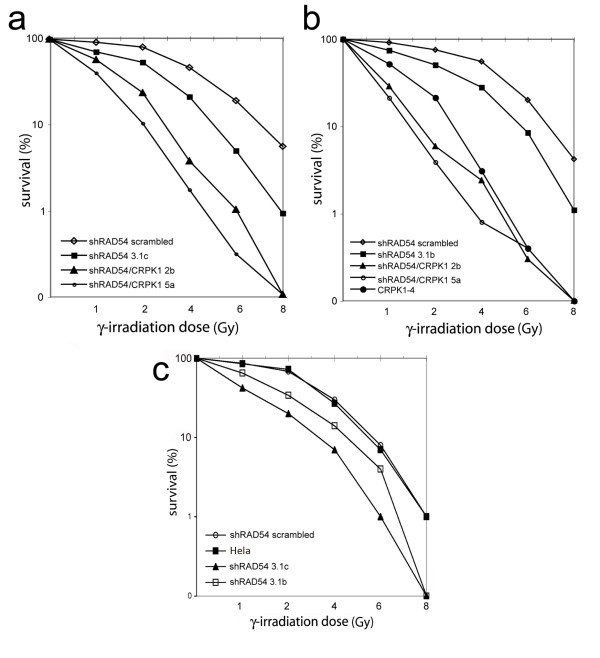
**Sensitivity to γ-irradiation**. The results of three different experiments (A, B and C) are reported in which the following cell lines were analyzed: single RAD54 inhibited (shRAD54 3.1c and shRAD54 3.1b), single DNA-PKcs inhibited (CRPK1-4) and double RAD54/DNA-PKcs (shRAD54/CRPK 2b and shRAD54/CRPK 5a) inhibited cell lines. The HeLa parental cell line and cell line stably transfected with the scrambled plasmid (shRAD54 scrambled) were used as control. Survival to irradiation for all cell lines was measured in duplicate samples using a clonogenic assay.

Since it is known that RAD54 defects cause radiation sensitivity [22], we measured the survival of the KD cell lines shRAD54 3.1c and shRAD54 3.1b to γ-irradiation at five different doses (1, 2, 4, 6 and 8 Gy; Figure 2). Both cell lines were hypersensitive to γ-irradiation (Lethal Dose or LD_50 _~ 2 Gy) compared to the control cell line containing the scrambled sequences and to HeLa cells (whose LD_50 _~ 4 Gy). The sensitivity to γ-rays in the RAD54 KD cell lines was less severe than in the previously isolated DNA-PKcs defective cell line CRPK1-4 (LD_50 _~ 1Gy) [14] while in the double KD clones (shRAD54/CRPK 2b and shRAD54/CRPK 5a) sensitivity was higher than in single KD cells (LD_50 _~ 0.5 Gy). In conclusion, impairment in the HR repair pathway causes hypersensitivity to γ-rays, and defects in both pathways further increase sensitivity.

### Frequency of generation of methotrexate resistant clones

MTX is a potent competitive inhibitor of the dihydrofolate reductase (DHFR) enzyme, which is essential for DNA synthesis and cell growth [28]. It is well known that mammalian cells have the capacity to acquire resistance to MTX through amplification of the *DHFR *gene, among other mechanisms.

Preliminary experiments were carried out to determine the sensitivity to MTX of the cell lines by measuring the inhibition of growth in massive cell cultures; the dose inhibiting cell growth to 50% (DR_50_) was found to be approximately 10 nM for all the cell lines (data not shown).

The frequency of MTX resistant colonies was then measured taking into account the plating efficiency of each cell line. The results of four experiments performed at two different MTX concentrations are shown in Table 1 and in Additional file 1. We detected a mild if any (2-3 fold) increment in the frequency of MTX resistant colonies in the RAD54 defective cell lines compared to the control cell lines (HeLa wt and pSSP-1, Table 1). Moreover, and according to our previous studies [14], cell lines defective for DNA-PKcs (CRPK1.1 and CRPK1.4, Figure 1c) showed a greater increment in the frequency of gene amplification (between 3.3 and 8 fold). Surprisingly and in spite of their radiosensitivity, the double KD cell lines (shRAD54/CRPK 2b and shRAD54/CRPK 5a) showed a frequency of MTX resistant colonies similar, or even lower in some experiments, compared to control cell lines (Table 1).

**Table 1:** Relative gene amplification ability

	Ratio *versus *HeLa parental line									
	
Cell lines	45 nM MTX					55 nM MTX				
	**1**	**2**	**3**	**4**	**Mean**	**1**	**2**	**3**	**4**	**Mean**

Hela	1	1	1	1	**1**	1	1	n.a	1	**1**

pSSP1	2.8	0.6	1.7	1.1	**1.5**	2.0	0.7	n.a.	0.9	**1.2**

CRPK 1.1	6.4	3.0	17.3	n.a.	**8.9**	3.5	3.1	n.a.	n.a.	**3.3**

CRPK 1.4	11.4	2.9	18.3	2.1	**8.7**	4.5	5.1	n.a.	4.5	**4.7**

shRAD54 3.1b	2.2	2.8	2.0	n.a.	**2.3**	2.0	2.1	n.a.	n.a	**2.0**

shRAD54 3.1c	5.0	1.4	3.0	3.4	**3.2**	3.0	1.1	n.a.	5.3	**3.1**

shRAD54/CRPK 2b	n.a.	0.3	1.1	0.8	**0.7**	n.a	0.3	n.a.	1.3	**0.8**

shRAD54/CRPK 5a	0.4	0.3	0.6	1.4	**0.7**	0.5	0.3	n.a.	1.2	**0.4**

Cell lines with different repair function impaired were analyzed together with their respective controls. The values represent the ratio between the frequency of MTX-resistant clones in each cell line *versus *the HeLa parental cell line in four different experiments and at two different MTX concentrations. n.a.: not-analyzed.

### Cytogenetic characterization of MTX-resistant clones

**Figure 3:**
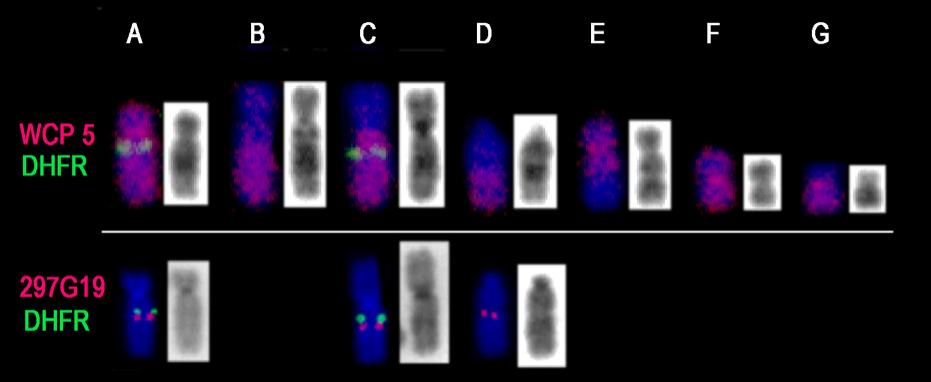
**Chromosome 5 complement of the HeLa parental cell line used for the construction of defective cell lines**. In all instances, the *DHFR *probe is labeled in green, whereas whole chromosome 5 painting and the BAC clone RP11-297G19 are labeled in red. FISH using the human chromosome 5 painting revealed the presence of seven chromosomes (A, B, C, D, E, F and G) with complete or partial homology to human chromosome 5. Chromosome A corresponds to a normal human chromosome 5 with a deletion of the chromosomal band 5q35; this organization was demonstrated by the lack of hybridization signal using the BAC clone RP11-117L6 (data not shown). Chromosome C corresponds to a translocated derivative chromosome between 5q and the short arm of chromosome 3 [der(3p5q)]. Chromosome B contains the region corresponding to 5q22.2-q35 translocated with an unknown chromosome, whereas chromosome D corresponds to 5q14-qter also translocated with another chromosome. In addition, chromosome F is an isochromosome of the p arm [i(5p)].

It is known that the *DHFR *gene is located on human chromosome 5q14. Since HeLa cells have an aneuploid and rearranged karyotype, we first characterized the chromosomes bearing the *DHFR *gene in the cell line used for the present studies. FISH analysis using a human chromosome 5 painting probe and a BAC containing the *DHFR *gene (CTC-325J23) revealed the presence of seven chromosomes (A, B, C, D, E, F and G) with complete or partial homology to human chromosome 5 (Figure 3); two of these chromosomes (A and C) carry one copy of the *DHFR *gene located in the proximal region of the long arm, corresponding to the 5q14 band, whereas the other markers derived from chromosome 5 (B, D, E, F and G) do not contain the *DHFR *gene. A BAC clone (RP11- 297G19), which maps to 5q15, was then hybridized with the chromosomes of HeLa cells together with the BAC containing the *DHFR *gene. The results showed that the RP11- 297G19 sequence is located on chromosomes A and C, near the *DHFR *gene, and on chromosome D, where the *DHFR *gene is absent (Figure 3).

To test whether MTX resistance was due to amplification of the *DHFR *gene, we isolated a total of 56 MTX resistant independent clones from different cell lines: six clones from parental HeLa cells, twelve from shRAD54 3.1c, twelve from shRAD54 3.1b, five from CRPK1.1, ten from CRPK1.4, six from shRAD54/CRPK 5a and five from shRAD54/CRPK 2b cells. All the clones were expanded to obtain clonal populations. Additionally, fourteen clones (five clones from parental cells and nine clones defective in DNA-PKcs) previously isolated in our laboratory [14] were included in the present study. Chromosome spreads were prepared at initial passages (passages 4-6) following clonal isolation and FISH experiments were performed hybridizing the DHFR BAC and the RP11- 297G19 BAC probes with all the clones. A detailed cytogenetic analysis revealed that gene amplification was the main mechanism responsible for MTX resistance in cell lines defective for DNA DSB repair mechanisms (Table 2). 67% (16 out 24) of the clones isolated from DNA-PKcs defective cells, 67% (16 out of 24) of those obtained from RAD54 defective cell lines and 64% (7 out of 11) of those obtained from the double defective RAD54/DNA-PKcs cells contained amplified DNA, while in only 36% (4 out of 11) of the clones isolated from parental HeLa cells gene amplification was detected. In order to shed light into the mechanisms responsible for amplification, we characterized in more details the chromosomal organization of the different amplicon structures observed. Regardless of the type of amplicon detected, all the clones analyzed contained two or more chromosomes carrying a single copy of the *DHFR *gene; when more than two *DHFR *carrying chromosomes were detected, the extra-chromosomes probably resulted from non-disjunction and might contribute to MTX resistance.

**Table 2:** Gene amplification analysis in MTX-resistant clones

			N° (%) MTX-resistant clones with					
			
Knocked-down gene	N° clones analyzed	N° amplified clones (%)	HSR	DMs	HSR DMs	i(5q)	DMs i(5q)	HSR DMs i(5q)
None (HeLa wt)	11	4 (36%)	0	4 (100%)	0	0	0	0
DNA-PKcs	24	16 (67%)	0	14 (87.5%)	1 (6.2%)	0	0	1 (6.2%)
RAD54	24	16 (67%)	1 (6.2%)	6 (37.5%)	3 (18.7%)	4 (25%)	2 (12.5%)	0
RAD54 and DNA-PKcs	11	7 (64%)	2 (28.6%)	1 (14.3%)	0	3 (42.8%)	1 (14.3%)	0

The clones analyzed derived from cell lines with different repair functions impaired. The organization of the *DHFR *gene was analyzed on mitotic chromosomes by FISH with a specific *DHFR *probe. HSR: homologous staining regions, DMs: double minutes, i(5q): isochromosome.

**Figure 4:**
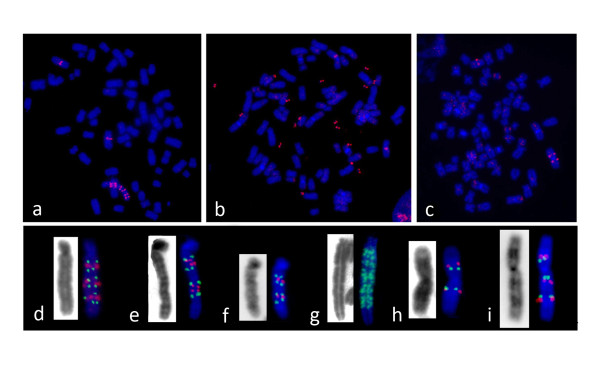
**Cytogenetic structure of DHFR gene amplicons**. *Upper section of the panel*: metaphase spreads hybridized with the BAC clone containing the *DHFR *gene (red signal) showing (**a**) a *DHFR *gene amplicon organized as a ladder, (**b**) DMs and (**c**) i(5q). *Lower section of the panel*: examples of different ladder-like structures (**d-g**) and i(5q) (**h-i**) found in MTX-resistant clones analyzed using double-colour FISH with the BAC containing the *DHFR *gene (green signal) and the BAC RP11-297G19 (red signal).

Interestingly, amplification of the *DHFR *gene was observed in different cytogenetic configurations depending of the genetic background of the cell line (Table 2 and Figure 4). We detected homogeneous staining regions (Figure 4a), extra-chromosomal double minutes (Figure 4b), isochromosomes 5q [i(5q)] (Figure 4c), as well as additional labeled chromosomes. DMs were the predominant amplified structures observed in MTX-resistant HeLa parental cell (100% of the clones analyzed) and DNA-PKcs defective clones (87.5%). Both DMs and HSR were observed in clones defective for RAD54 and/or DNA-PKcs proteins, while the i(5q) was observed almost exclusively in MTX-resistant clones defective for RAD54 (Table 2).

By double colour FISH with the *DHFR *and the RP11- 297G19 BAC clones we could detect different types of HSRs (Figure 4 d-g). The majority of these structures were clearly organized as inverted repeats (Figure 4 d-f), suggesting that successive BFB cycles, initiated by a DSB downstream from the 5q15 chromosomal band and followed by sister chromatid fusion, were the main mechanism of formation. In the unique DNA-PKcs defective clone with HSRs (CRPK1.1-45nM-9), as well as in one of the RAD54 deficient clones (shRAD54 3.1c-55nM-4), HSRs containing multiple copies of the *DHFR *gene organized as compact ladder-like structures without any hybridization signal for BAC RP11- 297G19 were observed (Figure 4g), suggesting that these structures could be composed by short inverted repeats, deriving from BFB cycles starting from a break very close to the *DHFR *gene, or by direct repeats. Both clones contained, either this type of HRS (22.7% of the cells analyzed in CRPK1.1-45nM-9 and 3.4% in shRAD54 3.1c-55nM-4) or DMs (60.6% of the cells analyzed in the clone defective for DNA-PKcs and 91.5% in the clone defective for RAD54) or both structures (4.5% in CRPK1.1-45nM-9 and 5.1% shRAD54 3.1c-55nM-4). The observation of HSRs of variable length suggests that chromosomal breakage occurred at different positions along the 5q arm; the presence of HRSs and DMs in different cells from the same clone and, in a few cases, in the same cell, suggests that breaks occurring within the amplified region following its formation could be responsible for the generation of DMs. To test this hypothesis, we propagated one of the cell lines (shRAD54 3.1c-55nM-4) for 30 additional passages and performed cytogenetic analysis again. We found that 100% of the cells contained only DMs, supporting the hypothesis that DMs derived from multiple breakages within HSR after long periods in culture.

i(5q) chromosomes carrying amplified DNA on both arms represented an additional type of marker chromosome indicating that isochromosome formation is an important mechanism by which HeLa cell lines developed resistance to MTX. FISH analyses revealed that, in all these chromosomes, the *DHFR *gene and the BAC clone RP11- 297G19 were organized symmetrically relative to the centromere (Figure 4h), as expected for isochromosomes. A remarkable result was the fact that isochromosomes were observed in MTX-resistant clones defective for RAD54 (with the exception of one clone defective for DNA-PKcs with only15% of the metaphases showing this structure). Six out of the sixteen (37.5%) amplified clones defective for RAD54 and four out of seven (57.1%) amplified clones derived from double defective cells presented the i(5q) (Table 2). In the majority of these clones, the i(5q) was the only structure bearing additional *DHFR *copies and was detected in 100% of the metaphases analyzed; in a few cases, it was detected in combination with DMs and/or HSR in the same cells (Table 2). It is important to note that i(5q) could undergo intra-chromosomal reorganizations in one of the double defective MTX-resistant clones, reflecting the dynamic nature of this structure. In Figure 4i an example of an i(5q) with a duplication of the chromosomal region bearing the *DHFR *gene is shown.

## Discussion

It is well known that several molecular mechanisms are implicated in gene amplification [5,29,30], all of which involve DNA DSBs as a primarily event (reviewed in [10]). We have previously shown that gene amplification occurs at higher frequency both in rodent [23] and HeLa [14] cells defective for NHEJ than in proficient cells. A possible explanation for this finding is that, in the absence of a functional NHEJ, the repair of broken DNA molecules is delayed, allowing misrepair of DNA ends and the initiation of gene amplification. Hinz and collaborators [31] have shown that also a defect in HR genes, such as *RAD51D*, *xrcc2 *and *xrcc3*, leads to an increased gene amplification ability in Chinese hamster cell lines (CHO).

In this study, we investigated the consequence of a defect in the *RAD54 *HR repair gene on gene amplification ability of human cells; in addition, we tested the effect of a combined deficiency in both HR and NHEJ. To this purpose, we constructed HeLa cell lines in which either *RAD54*, or both *RAD54 *and the NHEJ gene *DNA-PKcs*, had been stably knocked down by RNA interference. The DNA-PKcs knocked down cells were previously described in [14]. The successful impairment of *RAD54 *expression was demonstrated by the lower levels of the protein and by the greater sensitivity of knocked down cells to ionizing radiations compared to wild type cells. DNA-PKcs single deficient cell lines were more radiosensitive than RAD54 deficient cells supporting the hypothesis that the NHEJ mechanism plays a predominant role in the repair of radio-induced DSBs. Such radiosensitivity was enhanced in double KD RAD54/DNA-PKcs cells, revealing that the correct function of both repair mechanisms is essential for cell survival. These results extend those previously obtained by different authors showing that HR and NHEJ factors cooperate in the maintenance of genome stability in mammalian cells [32-34].

To study gene amplification ability of the cells KD in the different functions, we analyzed MTX resistance, which is frequently due to amplification of the *DHFR *gene. In the different cell lines, we determined the frequency of MTX resistant clones, the proportion of amplified clones among the resistant ones and the organization of the amplified DNA on mitotic chromosomes. The RAD54 deficient cell lines showed a frequency of MTX clones slightly higher than proficient cells, but lower than that observed in DNA-PKcs knocked down cells, while the proportion of amplified clones in the two deficient cell lines was similar (67%), and greater than that observed in HeLa cells (36%). These results suggest that, when the HR repair mechanism is impaired, the fraction of DSBs undergoing an improper repair and being engaged in gene amplification is lower than in cells deficient in NHEJ, but still higher than in proficient cells. This confirms again that the two repair mechanisms collaborate to prevent genome instability. In the cell lines deficient for both RAD54 and DNA-PKcs, the frequency of MTX resistant clones was lower than in proficient cells. Although we could still find amplified resistant clones, it is likely that when both the repair mechanisms are impaired, fewer cells can survive to the events involving DNA breakage that leads to gene amplification. The lack of increase in the frequency of amplified mutants in the double-deficient cells may derive from a balance between the increased generation of amplifications, due to the persistence of unrepaired DSBs, and the death of the cells that cannot survive to the breakage events required to trigger gene amplification, because of the severe DNA repair mechanism impairment.

In the amplified RAD54 deficient cells we found three major structures bearing the extra-copies of the *DHFR *gene: DMs, HSRs and isochromosomes of the long arm of chromosome 5, where the *DHFR *gene is located. Analysis of the organization of HSRs by double colour FISH with probes for the selected gene and for an adjacent region revealed that the amplicons were organized as inverted repeats, indicating that BBF cycles were the most likely mechanism of origin. It has been postulated that DMs can originate from breakages within HSRs [35,36]. In one clone, we could clearly demonstrate the transition from HSRs to DMs; in fact, at early stages after selection, both DMs and HSRs were detected in the MTX resistant clonal population, while after further propagation in culture, DMs remained the only structures bearing amplification.

Isochromosomes of the long arm of chromosome 5 were a peculiarity of the cells deficient for RAD54, either single KD or double KD for RAD54 and DNA-PKcs; in fact, they were present in about 40% of the MTX resistant clones isolated from these cells lines, while they were detected only in a minority of the mitoses of a single clone derived from DNA-PKcs deficient cells.

Although studies dealing with the molecular mechanisms responsible for the formation of isochromosomes are scarce, pericentromeric or centromeric breakage, followed by unequal recombination among highly repetitive sequences, have been hypothesized as possible causes of this cytogenetic abnormality [37-39]. Human centromeres contain extensive copies of repetitive elements called α-satellite [40] and it is well known that centromeres, jointly with telomeres, are regions of the chromosomes where recombination occurs very frequently [41,42]. Recently, Nakamura and co-workers [39] showed in the yeast *Schizoccharomyces pombe *that a failure in the HR mechanism, because of a defect in the RAD51 recombinase, increases the frequency of isochromosome formation. In yeast, DNA breaks within the centromere can be repaired through gene conversion, which depends on RAD51, or through break-induced replication (BIR), which is RAD51 independent. In the absence of a proficient recombination-mediated repair, DSB repair can preferentially occurs through BIR, which can lead to isochromosome formation when DNA synthesis is primed by an inverted repeated sequence that snaps back to align with its complementary sequence. Interestingly, it was shown [39] that RAD51 is associated with centromeres during the S phase, supporting the hypothesis that this protein can suppress the rearrangements of centromeric repeats that result in isochromosome formation. Tinline-Purvis and collaborators [43] found that defects in other HR functions increases the frequency of isochromosome formation. In addition, they demonstrated that also breaks far from the centromeres can lead to these chromosome anomalies, because of the occurrence of an extensive processing of DNA ends, when the efficiency of gene conversion is reduced.

## Concluding remarks

Our results indicate that a failure of HR moderately increases the frequency of gene amplification in human cells, suggesting that NHEJ is not sufficient to correctly repair all DNA breaks when HR is impaired. In addition, our observation of an increased frequency of isochromosome formation in HeLa cells deficient for RAD54 suggests that, similarly to what observed in fission yeast, also in human cells homologous recombination proteins, such as RAD54, can play a role in preventing rearrangements of the centromeres.

## Methods

### Cell culture and transfection

HeLa cells were routinely cultured at 37°C in 5% CO_2_, in DMEM supplemented with 10% fetal calf serum, glutamine and non-essential amino acids. During transfection and selection experiments 0.1 mg/ml penicillin and 100 U/ml streptomycin were added.

To inhibit *RAD54 *expression four plasmids containing the neomycin (G418) resistance gene with four different inserts for shRNA production under the control of the U1 promoter were used (SureSilencing™ kit, Cat. number KH01719N). The insert sequence of plasmids shRNAp-1, shRNAp-2, shRNAp-3 and shRNAp-4 were TCTCGtcaccagcattgtgaatagatCTTCCTGTCAatctattcacaatgctggtgaCT, TCTCGaaggttgtagaacgcttcaatCTTCCTGTCAattgaagcgttctacaaccttCT, TCTCGtgtggttgttgaccctattctCTTCCTGTCAagaatagggtcaacaaccacaCT and TCTCGcgagttgaaggagctgtttatCTTCCTGTCAataaacagctccttcaactcgCT, respectively. An additional plasmid (p-scrambled) containing a scrambled sequence, which is not homologous to any human gene (TCTCggaatctcattcgatgcatacCTTCCTGTCAgtatgcatcgaatgagattccCT) was used as control. To inhibit DNA-PKcs expression a plasmid previously described [14], containing the puromycin resistance gene and an oligonucleotide for the expression of an shRNA against DNA-PKcs, was used; the plasmid pSSP, not containing the oligonucleotide insert, was used to produce the control cell line pSSP1.Transfection experiments were carried out as previously described [44]. Briefly, 10^5 ^cells were seeded in complete medium in 10 cm dishes; after 24 h the medium was replaced with serum free medium and 1-5 μg of plasmid DNA in a 1 mM PEI (Polyethylenimine, Sigma) solution was added. After 7 h at 37°C, the medium was replaced with complete medium. After 24 h, selective medium containing 1 μg/ml puromycin or 800 μg/ml G418 was added. Puromycin and/or G418 resistant colonies were isolated after 4-5 weeks.

### Immunological detection of RAD54 and DNA-PKcs

For indirect immunofluorescence analysis of *RAD54 *and *DNA-PKcs *expression, cells were spread on slides with a cytospin centrifuge and permeabilized at 37°C for 15 minutes in KCM buffer (KCl 120 mM, NaCl 20 mM, Tris-HCl 10 mM, Na-EDTA 0.5 mM and Triton X-100 0.1% v/v). The slides were then incubated with either the anti-RAD54 (Abcam) or the anti-DNA-PKcs (Ab-4, Neomarkers) antibody diluted 1:200 in KCM containing 1% BSA at 37°C for 1 h. After washing with KB^- ^buffer (Tris-HCl 10 mM, NaCl 150 mM and BSA 1%) the slides were treated with a goat anti-mouse texas-red conjugated secondary antibody (Jackson ImmunoResearch) at 37°C for 30 min. The cells were washed again with KB^- ^buffer, stained with DAPI (4, 6-diamidino-2-phenylindole) and visualized with a ZEISS Axioplan fluorescence microscope. Images were captured with a Photometric CCD camera and processed with the IP-Lab software.

To obtain total cell extracts for Western blots the cells were washed twice with cold PBS, resuspended in Laemmli buffer and boiled for 10 min. Proteins were separated by SDS-PAGE gel electrophoresis and electroblotted to nitrocellulose membranes (Amersham). The membranes were then incubated with the antibody against DNA-PKcs diluted 1:3000 or with the antibody against RAD54 diluted 1:100; and with the antibody against tubulin (NeoMarkers), diluted 1:3000. A goat antimouse monoclonal (Pierce), diluted 1:5000 was used as secondary antibody. The pre-incubation of the membranes and the dilution of all antibodies were performed in 1xPBS containing 0,05% Tween20 and 7.5% non-fat dry milk. The signals detection was performed by chemiluminescence (Immun-Star WesternC Kit, BioRad).

### Sensitivity to irradiation

Irradiation was carried out using a ^60^Co-ray source at a dose rate of 1.3 Gy/min. Exponentially growing cells were tripsinized, resuspended in complete medium and irradiated. After irradiation, the cells were diluted and seeded in 10 cm plates (500 cells/plate). After 10 days, colonies were fixed with methanol, stained with Coomassie Blue (1% Page Blue in 50% methanol and 7.5% acetic acid) and the number of colonies with more than 50 cells was counted. The γ-ray dose reducing survival to 50% (LD_50_) was then calculated after plotting survival against γ-ray doses.

### Sensitivity to methotrexate and analysis of gene amplification frequency

Sensitivity to methotrexate (MTX) was determined by measuring the inhibition of growth in massive cell cultures. Samples of 2 × 10^5 ^cells were plated in 6 cm dishes in complete medium containing different MTX concentrations. After 96 h at 37°C, the cells were tripsinized, centrifuged, washed in PBS and centrifuged again. The pellets were dissolved in 0.1 M NaOH for 30 min at 50°C; absorbance of cellular lysates was measured at 260 nm. The drug concentration inhibiting cell growth by 50% (Dose Response 50 or DR_50_) was determined from a plot of absorbance against drug concentration. To measure gene amplification frequency, samples of 10^5 ^cells were seeded in selective medium containing two different MTX concentrations, 45 nM and 55 nM MTX (4.5 and 5.5 times the LD_50_). In addition to two single RAD54 defective and two double RAD54/DNA-PKcs defective cell lines, we included in the experiments the two previously isolated DNA-PKcs defective cell lines CRPK1.1 and CRPK1.4 [14] and their control cell line (a clone from HeLa cells transfected with the empty plasmid pSSP, named pSSP-1). Five samples were plated per each drug concentration. After 2 weeks, the surviving MTX resistant colonies were fixed, stained and counted. The mutation frequency was calculated from the mean number of colonies among the cultures [26]. For each cell line, the mean was corrected for the plating efficiency, determined by counting the number of colonies recovered after seeding 500 cells in 10 cm dishes.

### Cytogenetic analysis

To detect the presence of the *DHFR *gene, fluorescence in situ hybridizations (FISH) was performed using the BAC clone CTC-325J23 (CalTech human BAC library, AC008434). The chromosome 5 specific BAC probe used (RP11- 297G19) was obtained from the RPCI-11 human bacterial artificial chromosome (BAC) library. The whole-chromosome 5 painting probe derives from flow sorted human chromosomes. Chromosome spreads and FISH were performed as previously described [45]. Briefly, probes were labeled by nick translation with Cy3-dUTP and Alexa-dUTP and chromosomes were stained with DAPI (4, 6-diamidino-2-phenylindole). Digital images were captured as indicated above.

## Competing interests

The authors declare that they have no competing interests.

## Authors' contributions

ARH: research design, performed experiments, data analysis, manuscript writing. AS: performed experiments, data analysis. LK: performed experiments, data analysis. SGN: performed experiments, data analysis. CM: data analysis, manuscript writing. EG: study conception, research design, data analysis, manuscript writing. All authors have read and approved the final manuscript.

## Supplementary Material

Additional file 1 **Number of methotrexate (MTX) resistant colonies in RAD54 and DNA-PKcs defective cell lines**. Mean number of MTX resistant colonies per plate in cell lines with different repair functions impaired for each experiment, including standard deviations. Click here for file
