# Supplementary material for: Gene amplification in human cells knocked down for RAD54
Source: Genome Integr. 2011 Mar 18;2:5. doi: 10.1186/2041-9414-2-5 (PMC3074559; doi:10.1186/2041-9414-2-5)
Supplement: Additional file 1 — Number of methotrexate (MTX) resistant colonies in RAD54 and DNA-PKcs defective cell lines. Mean number of MTX resistant colonies per plate in cell lines with different repair functions impaired for each experiment, including standard deviations. [file 2041-9414-2-5-S1.DOC]

**Additional File 1. Number of methotrexate resistant colonies in RAD54 and DNA-PKcs defective cell lines**

| **Cell line** | **[MTX] nM** | **Mean number of MTX resistant colonies ± SD** | **Ratio *versus* HeLa parental line** |
| --- | --- | --- | --- |
| **Experiment 1** | | | |
| HeLa wt | **45**  **55** | 5 ± 2,3  2 ± 0,6 | **1,0**  **1,0** |
| pSSP1 | **45**  **55** | 14 ± 6,5  4 ± 2,1 | **2,8**  **2,0** |
| CRPK1.1 | **45**  **55** | 32 ± 6,5  7 ± 2,5 | **6,4**  **3,5** |
| CRPK1.4 | **45**  **55** | 57 ± 7,8  9 ± 5,5 | **11,4**  **4,5** |
| shRAD54 3.1b | **45**  **55** | 11 ± 3,8  4 ± 1,0 | **2,2**  **2,0** |
| shRAD54 3.1c | **45**  **55** | 25 ± 3,2  6 ± 0,6 | **5,0**  **3,0** |
| shRAD54/CRPK 2b | **45**  **55** | n.a.  n.a. | **n.a**  **n.a** |
| shRAD54/CRPK 5a | **45**  **55** | 2 ± 1,0  1 ± 1,2 | **0,4**  **0,5** |
| **Experiment 2** | | | |
| HeLa wt | **45**  **55** | 31 ± 7,6  7 ± 2,5 | **1,0**  **1,0** |
| pSSP1 | **45**  **55** | 20 ± 4,3  5 ± 2,2 | **0,6**  **0,7** |
| CRPK1.1 | **45**  **55** | 94 ± 12,1  22 ± 3,1 | **3,0**  **3,1** |
| CRPK1.4 | **45**  **55** | 91 ± 6,7  36 ± 4,7 | **2,9**  **5,1** |
| shRAD54 3.1b | **45**  **55** | 87 ± 3,2  15 ± 2,2 | **2,8**  **2,1** |
| shRAD54 3.1c | **45**  **55** | 43 ± 3,3  8 ± 2,3 | **1,4**  **1,1** |
| shRAD54/CRPK 2b | **45**  **55** | 9 ± 2,4  2 ± 0,8 | **0,3**  **0,3** |
| shRAD54/CRPK 5a | **45**  **55** | 10 ± 2,9  2 ± 0,9 | **0,3**  **0,3** |
| **Experiment 3** | | | |
| HeLa wt | **45**  **55** | 3 ± 2,3  n.a. | **1,0**  **n.a.** |
| pSSP1 | **45**  **55** | 5 ± 2,0  n.a. | **1,7**  **n.a.** |
| CRPK1.1 | **45**  **55** | 52 ± 13,6  n.a. | **17,3**  **n.a.** |
| CRPK1.4 | **45**  **55** | 55 ± 9,6  n.a. | **18,3**  **n.a.** |
| shRAD54 3.1b | **45**  **55** | 6 ±2,7  n.a. | **2,0**  **n.a.** |
| shRAD54 3.1c | **45**  **55** | 9 ± 2,5  n.a. | **3,0**  **n.a.** |
| shRAD54/CRPK 2b | **45**  **55** | 3 ±1,3  n.a. | **1,1**  **n.a.** |
| shRAD54/CRPK 5a | **45**  **55** | 2 ± 1,0  n.a. | **0,6**  **n.a.** |
| **Experiment 4** | | | |
| HeLa wt | **45**  **55** | 122 ± 11,3  15 ± 3,1 | **1,0**  **1,0** |
| pSSP1 | **45**  **55** | 134 ± 14,8  13 ± 5,1 | **1,1**  **0,9** |
| CRPK1.1 | **45**  **55** | n.a.  n.a. | **n.a.**  **n.a.** |
| CRPK1.4 | **45**  **55** | 254 ± 14,1  67 ± 9,6 | **2,1**  **4,5** |
| shRAD54 3.1b | **45**  **55** | n.a.  n.a. | **n.a.**  **n.a.** |
| shRAD54 3.1c | **45**  **55** | 412 ± 13,1  78 ± 15,9 | **3,4**  **5,3** |
| shRAD54/CRPK 2b | **45**  **55** | 95 ± 5,6  19 ± 5,4 | **0,8**  **1,3** |
| shRAD54/CRPK 5a | **45**  **55** | 170 ± 15,8  18 ± 2,4 | **1,4**  **1,2** |
